# Evaluating the Impact of the Grading and Assessment of Predictive Tools Framework on Clinicians and Health Care Professionals’ Decisions in Selecting Clinical Predictive Tools: Randomized Controlled Trial

**DOI:** 10.2196/15770

**Published:** 2020-07-09

**Authors:** Mohamed Khalifa, Farah Magrabi, Blanca Gallego Luxan

**Affiliations:** 1 Centre for Health Informatics, Australian Institute of Health Innovation, Faculty of Medicine and Health Sciences Macquarie University Sydney Australia; 2 Centre for Big Data Research in Health Faculty of Medicine University of New South Wales Sydney Australia

**Keywords:** clinical prediction rule, clinical decision rules, evidence-based medicine, evaluation study

## Abstract

**Background:**

While selecting predictive tools for implementation in clinical practice or for recommendation in clinical guidelines, clinicians and health care professionals are challenged with an overwhelming number of tools. Many of these tools have never been implemented or evaluated for comparative effectiveness. To overcome this challenge, the authors developed and validated an evidence-based framework for grading and assessment of predictive tools (the GRASP framework). This framework was based on the critical appraisal of the published evidence on such tools.

**Objective:**

The aim of the study was to examine the impact of using the GRASP framework on clinicians’ and health care professionals’ decisions in selecting clinical predictive tools.

**Methods:**

A controlled experiment was conducted through a web-based survey. Participants were randomized to either review the derivation publications, such as studies describing the development of the predictive tools, on common traumatic brain injury predictive tools (control group) or to review an evidence-based summary, where each tool had been graded and assessed using the GRASP framework (intervention group). Participants in both groups were asked to select the best tool based on the greatest validation or implementation. A wide group of international clinicians and health care professionals were invited to participate in the survey. Task completion time, rate of correct decisions, rate of objective versus subjective decisions, and level of decisional conflict were measured.

**Results:**

We received a total of 194 valid responses. In comparison with not using GRASP, using the framework significantly increased correct decisions by 64%, from 53.7% to 88.1% (88.1/53.7=1.64; *t_193_*=8.53; *P*<.001); increased objective decision making by 32%, from 62% (3.11/5) to 82% (4.10/5; *t_189_*=9.24; *P*<.001); decreased subjective decision making based on guessing by 20%, from 49% (2.48/5) to 39% (1.98/5; *t_188_*=−5.47; *P*<.001); and decreased prior knowledge or experience by 8%, from 71% (3.55/5) to 65% (3.27/5; *t_187_*=−2.99; *P*=.003). Using GRASP significantly decreased decisional conflict and increased the confidence and satisfaction of participants with their decisions by 11%, from 71% (3.55/5) to 79% (3.96/5; *t_188_*=4.27; *P*<.001), and by 13%, from 70% (3.54/5) to 79% (3.99/5; *t_188_*=4.89; *P*<.001), respectively. Using GRASP decreased the task completion time, on the 90th percentile, by 52%, from 12.4 to 6.4 min (*t_193_*=−0.87; *P*=.38). The average System Usability Scale of the GRASP framework was very good: 72.5% and 88% (108/122) of the participants found the GRASP useful.

**Conclusions:**

Using GRASP has positively supported and significantly improved evidence-based decision making. It has increased the accuracy and efficiency of selecting predictive tools. GRASP is not meant to be prescriptive; it represents a high-level approach and an effective, evidence-based, and comprehensive yet simple and feasible method to evaluate, compare, and select clinical predictive tools.

## Introduction

### Background

Clinical decision support (CDS) systems have been discussed to enhance evidence-based practice and support cost-effectiveness [1-10]. On the basis of the three-level classification by Shortliffe, clinical predictive tools, referred to as predictive tools in this paper, belong to the highest CDS level, providing patient-specific recommendations based on clinical scenarios, which usually follow clinical rules and algorithms, a cost-benefit analysis, or clinical pathways [11,12]. Such tools include various applications, ranging from the simplest manual clinical prediction rules to the most sophisticated machine learning algorithms [13,14]. These research-based applications provide diagnostic, prognostic, or therapeutic decision support. They quantify the contributions of relevant patient characteristics to derive the likelihood of diseases, predict their courses and possible outcomes, or support decision making on their management [15,16].

When selecting predictive tools for implementation in clinical practice or for recommendation in clinical guidelines, clinicians and health care professionals, referred to as *professionals* in this paper, involved in decision making are challenged with an overwhelming and ever-growing number of tools. Many of these tools have never been implemented or evaluated for comparative effectiveness [17-19]. By definition, health care professionals include all clinicians who provide direct care to patients, in addition to professionals who work in laboratories, researchers, and public health experts [20]. Professionals usually rely on previous experience, subjective evaluation, or recent exposure to predictive tools in making selection decisions. Objective methods and evidence-based approaches are rarely used in such decisions [21,22]. When developing clinical guidelines, some professionals search the literature for studies that describe the development, implementation, or evaluation of predictive tools. Others look for systematic reviews comparing the tools’ performance or development methods. However, there are no available approaches to objectively summarize or interpret such evidence [23,24]. In addition, predictive tool selection decisions are time-consuming as they seek a consensus of subjective expert views [25]. Furthermore, when experts make their decisions subjectively, they face much decisional conflict; they are less confident in the decisions they make and sometimes less satisfied with them [26].

To overcome this major challenge, the authors developed and published a new evidence-based framework for grading and assessment of predictive tools (the GRASP framework) [27]. The authors have also validated and updated the GRASP framework through the feedback of a wide group of international experts [28]. Furthermore, the authors applied the GRASP framework to evaluate and compare 14 pediatric head injury clinical predictive tools. This study is now published [29]. The GRASP framework aims to provide standardized objective information on predictive tools to support the search for and selection of effective tools. On the basis of the critical appraisal of published evidence, GRASP uses 3 dimensions to grade clinical predictive tools: (1) phase of evaluation, (2) level of evidence, and (3) direction of evidence.

### Phase of Evaluation

Predictive tools are assigned the letters A, B, or C based on the highest phase of evaluation: before implementation, during planning for implementation, or after implementation respectively. If a tool’s predictive performance, as reported in the literature, has been tested retrospectively for validity using observational data, it is assigned phase C. If a tool’s usability or potential effect have been tested prospectively using small pilots, which may or may not reflect routine practice, it is assigned phase B. Potential effect of a tool is the expected, estimated, or calculated impact of using the tool, assuming it has been successfully implemented and used in clinical practice. Finally, if a tool has been implemented in clinical practice and there is published evidence evaluating its achieved postimplementation impact prospectively, it is assigned phase A.

### Level of Evidence

A numerical score within each phase is assigned based on the level of evidence associated with each tool. A tool is assigned grade C1 if it has been tested for external validity multiple times, grade C2 if it has been tested for external validity only once, and grade C3 if it has been tested only for internal validity. Grade C0 means that the tool did not show sufficient internal validity to be used in clinical practice. Grade B1 is assigned to a predictive tool that has been evaluated during the planning for implementation, for both of its potential effect, on clinical effectiveness, patient safety, or health care efficiency, and for its usability. Grade B2 is assigned to a predictive tool that has been evaluated only for its potential effect, while if it has been studied only for its usability, it is assigned grade B3. Finally, if a predictive tool had been implemented and evaluated for its postimplementation impact on clinical effectiveness, patient safety, or health care efficiency, then it is assigned grade A1 if there is at least one experimental study of good quality evaluating its postimplementation impact, grade A2 if there are observational studies evaluating its impact, and grade A3 if the postimplementation impact has been evaluated only through subjective studies, such as expert panel reports.

### Direction of Evidence

For each phase and level of evidence, a direction of evidence is assigned based on the collective conclusions reported in the studies. The evidence is considered positive if all studies about a predictive tool reported positive conclusions and negative if all studies reported negative or equivocal conclusions. The evidence is considered mixed if some studies reported positive results, while others reported either negative or equivocal conclusions. To determine the overall direction of evidence, a protocol is used to sort the mixed evidence to support an overall positive or negative conclusion. The protocol is based on 2 main criteria: (1) degree of matching between the evaluation study conditions and the original tool specifications and (2) quality of the evaluation study. Studies evaluating tools in closely matching conditions to the tools’ specifications and providing high-quality evidence are considered first for their conclusions in deciding the overall direction of evidence.

**Figure 1:**
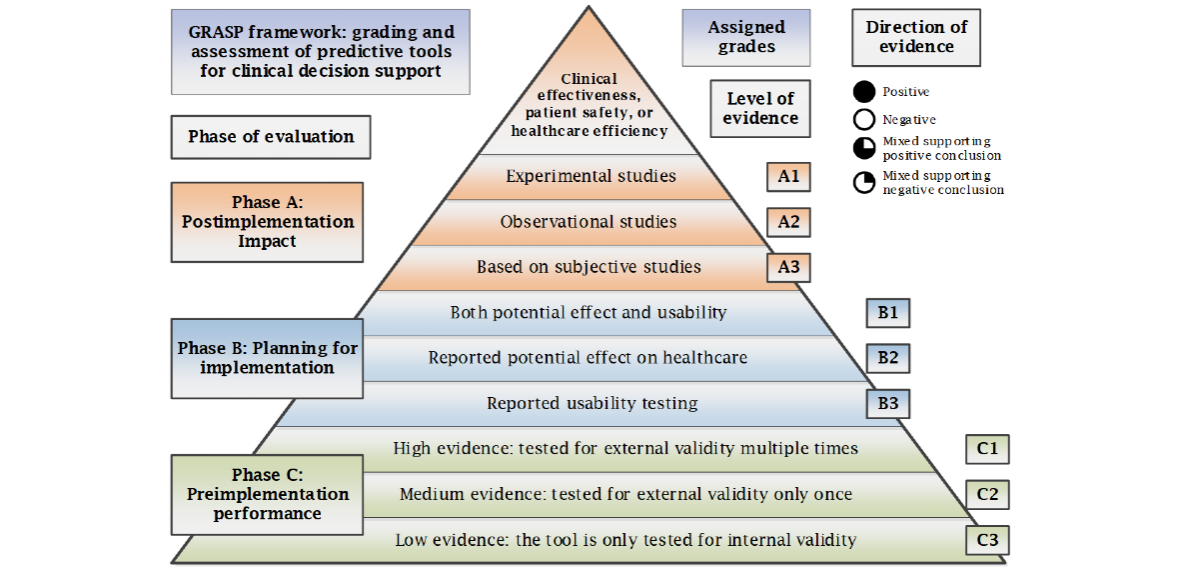
The grading and assessment of predictive tools framework concept.

The final grade assigned to a predictive tool is based on the highest phase of evaluation, supported by the highest level of positive evidence, or mixed evidence that supports a positive conclusion. More details on the process of critical appraisal of published evidence, summarizing the evidence, and assigning grades to predictive tools are discussed in a published study that describes the development of the GRASP framework [27]. The GRASP framework concept is shown in Figure 1, and a detailed report of the GRASP framework is presented in Multimedia Appendix 1.

The aim of this study was to evaluate the impact of using GRASP on the decisions made by professionals in selecting predictive tools for CDS. The objective was to explore whether the GRASP framework positively supports professionals’ evidence-based decision making and improves their accuracy and efficiency in selecting clinical predictive tools. To explore this impact, a group of hypotheses have been proposed including that using the GRASP framework by professionals is going to (1) make their decisions more accurate, that is, selecting the best predictive tools; (2) make their decisions more objective, informed, and evidence-based, that is, decisions are based on the information provided by the framework; (3) make their decisions less subjective, that is, decisions are less based on guessing, prior knowledge, or experience; (4) make their decisions more efficient, that is, decisions are made in less time; and (5) make them face less decisional conflict, that is, become more confident in their decisions and more satisfied with them. We also proposed that using GRASP can move professionals who have less knowledge, less experience, and are less familiar with predictive tools to an equal or even higher accuracy of decision making than professionals who have more knowledge, have more experience, and are more familiar with tools when they do not use GRASP.

## Methods

### The Study Design

This study was based on experimental methods. It aimed to examine the performance and outcomes of professionals’ decisions in selecting predictive tools with and without using the GRASP framework. Through a web-based survey, the experiment involved asking participants to select the best predictive tool for implementation in clinical practice or for recommendation in clinical practice guidelines from a group of 5 similar tools doing the same predictive task, one time with and another time without using the GRASP framework. In addition, participants were asked a few questions regarding the process of making their decisions through the 2 scenarios. Participants were also requested to provide their feedback on the perceived usability and usefulness of the evidence-based summary of the GRASP framework. This experiment does not include registration of the randomized controlled trial as it does not involve any patients, medications, or treatments.

**Figure 2:**
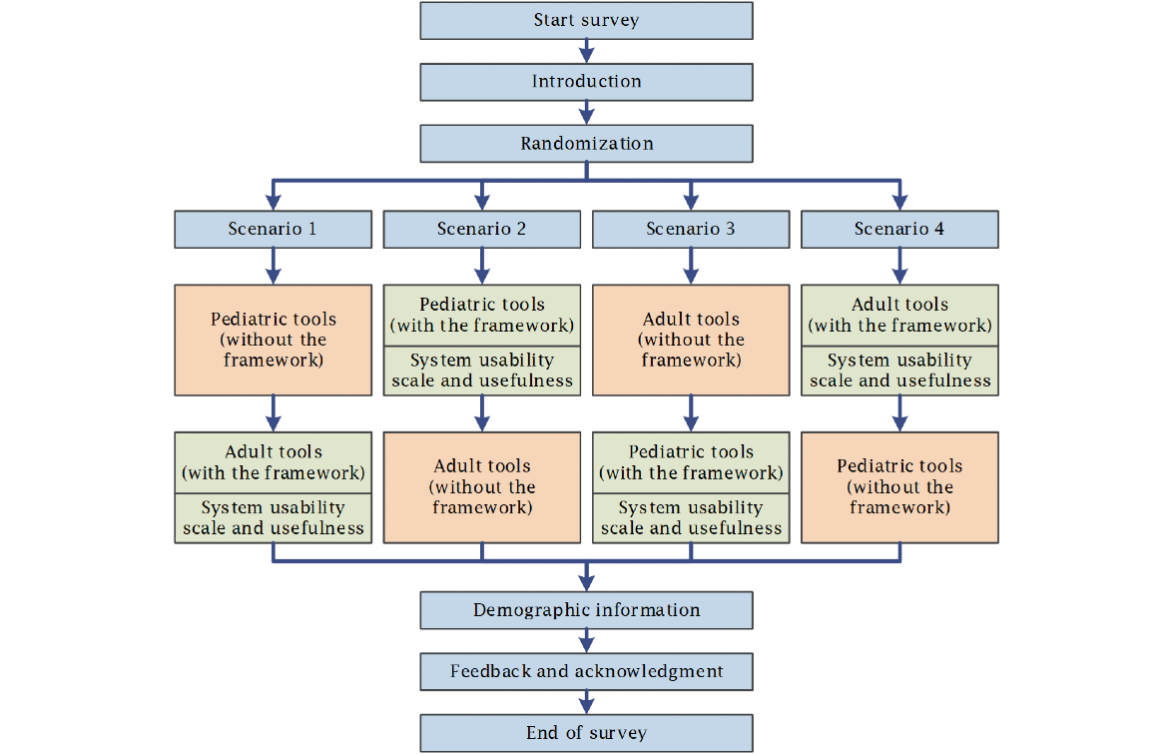
Survey workflow and randomization of the 4 scenarios.

The emergency department (ED) is among the top health care specialties that are increasingly utilizing predictive tools, especially in the area of managing traumatic brain injury (TBI), which is the leading cause of death and disability among trauma patients [30-33]. Two groups of predictive tools designed to exclude TBI in the ED were prepared. The first group included 5 tools for predicting TBI in pediatrics: Paediatric Emergency Care Applied Research Network (PECARN) head injury rule, Children's Head injury ALgorithm for the prediction of Important Clinical Events, Canadian Assessment of Tomography for Childhood Head injury rule, Palchak head injury rule, and Atabaki head injury rule [34-38]. The PECARN, being the most validated and the only tool that has been implemented in clinical practice and discussed to have a positive postimplementation impact, is the best tool among the 5 [39,40]. The second group includes 5 tools for predicting TBI in adults: the Canadian CT Head Rule (CCHR), New Orleans Criteria (NOC), Miller criteria for head computed tomography, Kimberley Hospital Rule, and Ibanez model for head computed tomography [41-45]. The CCHR and NOC, being the only tools that have been implemented in clinical practice and are the most validated, showing high predictive performance, are the best tools among the 5 [46-48]. Two scenarios were prepared for this experiment. The first is the control scenario, which includes providing participants with basic information about each tool, the full text of the original studies describing the tools, and allowing them to search the internet for information. The second is the experiment scenario, including providing participants with the main component of the GRASP framework, which is an evidence-based summary of the predictive tools and the full GRASP report on each tool, in addition to allowing them to search the internet for information. To minimize bias, eliminate the pre-exposure effect, and improve the robustness, the experiment includes randomizing the 2 groups of predictive tools and the 2 scenarios. Accordingly, the participants go randomly through 1 of 4 scenarios: (1) pediatric tools without GRASP and then adult tools with GRASP, (2) pediatric tools with GRASP and then adult tools without GRASP, (3) adult tools without GRASP and then pediatric tools with GRASP, and (4) adult tools with GRASP and then pediatric tools without GRASP. Figure 2 shows the survey workflow and the randomization of participants.

**Figure 3:**
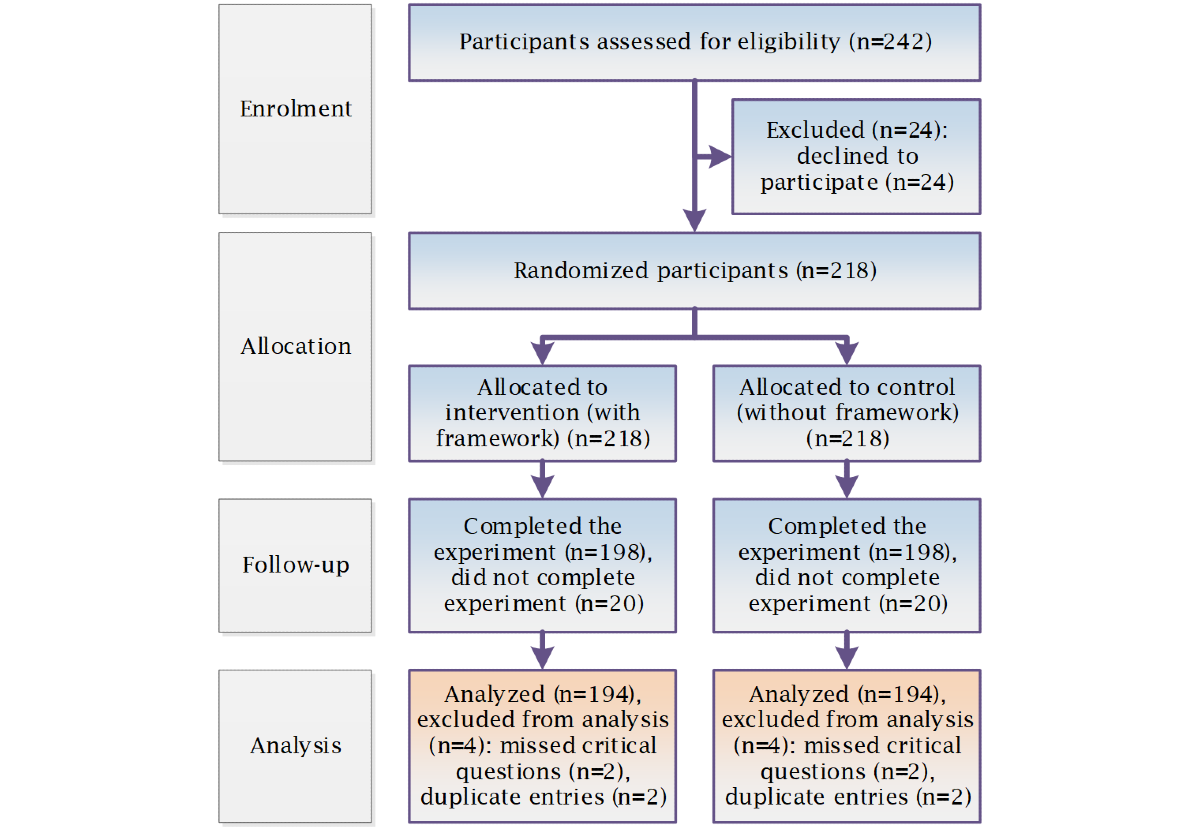
Consolidated Standards of Reporting Trials 2010 flow diagram of the experiment. GRASP: grading and assessment of predictive tools.

The authors recruited a wide group of international professionals to participate in this experiment through a web-based survey. To identify potential participants who work at the ED and those who have knowledge or experience about CDS tools, published studies were used to retrieve the authors’ emails and invite them. To retrieve studies on CDS systems, tools, models, algorithms, and pathways or rules used in the ED, emergency service, or emergency medicine published over the last 5 years by professionals who work in the EDs or services of their health care organizations or those who conducted emergency medicine, EDs, or emergency services research, 4 databases were used: MEDLINE, EMBASE, CINAHL, and Google Scholar. The authors expected a response rate of approximately 10%. Before the deployment of the survey, a pilot test was conducted by 10 expert professionals. The feedback of the pilot test was used to improve the survey. Professionals who participated in the pilot test were excluded from participation in the final survey. An invitation email, introducing details about the study objectives, the GRASP framework, the experiment task, the survey completion time, which was estimated at 20 min, and a participation consent was submitted to the identified potential participants with the link to the web-based survey. A reminder email, in 2 weeks, was sent to the potential participants who did not respond or complete the survey. Figure 3 shows the CONSORT 2010 flow diagram of the progress of the randomized trial of the 2 groups: intervention group (GRASP) and control group (No GRASP), showing the enrollment, intervention allocation, follow-up, and data analysis [49,50].

### The Study Survey

The web-based survey was developed using the Qualtrics Experience Management Solutions Platform [51]. The survey, illustrated through screenshots in the Multimedia Appendix 1, includes 5 sections. The first section includes an introduction to the study objectives, the GRASP framework, and the experiment task. In addition, participants are provided with contacts to request further information or submit complaints. The second section includes randomizing the 2 scenarios and the 2 groups of predictive tools to create the 4 scenarios described above.

In this section, participants are asked to assume that they are the heads of busy EDs and are responsible for selecting the best tool, the most validated in the literature or implemented in clinical practice, out of the 5 diagnostic head injury predictive tools. The PECARN is the correct answer among the 5 pediatric tools, and both the CCHR and the NOC are correct answers among the 5 adult tools. On a 5-point Likert scale, participants were asked to show how much they agreed to the following: (1) they made their decisions based on guessing, (2) they made their decisions based on prior knowledge or experience, (3) they made their decisions based on the information provided in the survey, (4) they were confident in their decisions, and (5) they were satisfied with their decisions. The third section includes asking participants to provide their feedback on the usability of the evidence-based summary of the GRASP framework through a standard set of System Usability Scale (SUS) questions. The SUS is a simple, 10-item attitude Likert scale that provides a global view of subjective assessments of usability. It was developed by John Brooke in 1986 as a tool to be used in the engineering of electronic systems. It is now widely accepted as a tool for the evaluation of system usability [52,53]. Participants were also asked to provide free-text feedback on whether they think the framework is useful or not and why they think so. The fourth section includes participants’ demographics, such as their clinical or health care role, specialty, gender, age group, years of experience, and how much they are familiar with head injury predictive tools.

### The Study Sample Size

As an initial estimate of the required sample size for this study, and based on similar studies, evaluating the impact of using information systems on professionals’ objective, informed, and evidence-based decisions, the authors aimed to recruit a sample of 40 to 60 participants [54-56]. More specifically, it was estimated that a sample size of 46 participants would be sufficient to test for at least a 10% difference, between the 2 arms of the experiment, in the measured outcomes, when using a paired two-tailed *t* test with a significance level of 0.05 and power of 0.95. Calculations were conducted using G*Power software [57].

### Analysis and Outcomes

To test the 5 proposed hypotheses, the study was designed to compare the 2 scenarios, making decisions with and without using the GRASP framework, based on a group of 7 measures: (1) time needed for tool selection decision making; (2) accuracy of tool selection decisions; (3) making decisions subjectively based on guessing; (4) making decisions subjectively based on prior knowledge or experience; (5) making decisions objectively based on the information and evidence provided; (6) levels of participants’ confidence in their decisions; and (7) levels of participants’ satisfaction with their decisions. The accuracy of making decisions, with and without GRASP, was also compared along with knowledge, experience, and familiarity with predictive tools. Table 1 shows the 5 proposed hypotheses and their related 7 outcome measures. To avoid an inflated Type I error and account for the 5 tested hypotheses and the 7 compared measures, the Bonferroni correction was used, by setting the alpha value of the paired samples *t* test to .007 instead of .05. The sample size was re-estimated to 96 participants. In addition, the SUS was calculated for the average rate and distribution of scores. The perceived usefulness and free-text feedback were analyzed. The demographic variables were analyzed for possible correlations or differences.

As this randomized controlled experiment was conducted via a web-based survey of clinicians and health care professions, Table 2 shows the checklist for reporting results from the internet surveys (checklist for reporting results from the internet surveys).

**Table 1:** Proposed hypotheses and related outcome measures.

Proposed hypotheses	Related outcome measures
Using GRASP^a^ will make predictive tools’ selection decisions more accurate, that is, selecting the best predictive tools	Accuracy of tools’ selection decisions
Using GRASP will make decisions more objective, informed, and evidence-based, that is, decisions are based on the information provided by the framework	Making decisions objectively based on the information and evidence provided in the experiment
Using GRASP will make decisions less subjective, that is, less based on guessing, prior knowledge, or experience	Making decisions subjectively based on guessing and making decisions subjectively based on prior knowledge or experience
Using GRASP will make decisions more efficient, that is, decisions are made in less time	The time needed for tools’ selection decision making
Using GRASP will make participants face less decisional conflict, that is, be more confident and satisfied with decisions	Levels of participants’ confidence in their decisions and levels of participants’ satisfaction with their decisions

^a^GRASP: grading and assessment of predictive tools.

**Table 2:** The checklist for reporting results from the internet surveys checklist.

Item category and checklist item		Explanation
**Design**		
	Describe survey design	A randomized controlled trial experiment testing the impact of using the GRASP framework on clinicians and health care professionals’ decisions in selecting predictive tools for CDS^a^, using a convenience invited sample to participate in the experiment
**IRB^b^ approval and informed consent process**		
	IRB approval	The experiment was approved by the Human Research Ethics Committee, Faculty of Medicine and Health Sciences, Macquarie University, Sydney, Australia
	Informed consent	Informed consent was introduced at the beginning of the survey for participants to agree before they take the survey, including the length of time of the survey, types of data collected and its storage, investigators, and the purpose of the study
	Data protection	Collected personal information was protected through Macquarie University account on Qualtrics survey system
**Development and pretesting**		
	Development and testing	The first author (MK) developed the survey and pilot tested the questions and its usability before deploying the survey to the participants
**Recruitment process and description of the sample having access to the questionnaire**		
	Open survey versus closed survey	This was a closed survey; only invited participants had access to complete the survey
	Contact mode	An initial contact, via email, was sent to all invited participants. Only those who agreed to participate completed the web-based survey
	Advertising the survey	The survey was not advertised. Only invited participants were informed of the study and completed the survey
**Survey administration**		
	Web or email	The survey was developed using the Qualtrics survey platform, and the link to the web-based survey was sent to invited participants via email. Responses were automatically collected through the Qualtrics survey platform then retrieved by the investigators for analysis
	Context	Only invited participants were informed of the study via email
	Mandatory/voluntary	The survey was not mandatory for invited participants
	Incentives	The only incentive was that participants could request to be acknowledged in the published study. Participants were also informed of the results of the survey after the analysis is complete
	Time/date	Data were collected over 6 weeks, from March 11 to April 21, 2019
	Randomization of items or questionnaires	To prevent biases, items were randomized. Figure 2 shows the survey workflow and randomization of 4 scenarios
	Adaptive questioning	Four scenarios were used and randomized, but they were not conditionally displayed
	Number of items	From 5 to 8 items per page
	Number of screens (pages)	The questionnaire was distributed over 5 pages
	Completeness check	Completeness checks were used after the questionnaire was submitted, and mandatory items were highlighted. Items provided a nonresponse option “not applicable” or “don’t know”
	Review step	Respondents were able to review and change their answers before submitting their answers
**Response rates**		
	Unique site visitor	We used the IP^c^ addresses to check for unique survey visitors
	View rate (ratio of unique survey visitors/unique site visitors)	Only invited participants had access to the survey. Survey visitors included those who completed the survey and those who started the survey but did not complete it or gave incomplete answers
	Participation rate (ratio of unique visitors who agreed to participate/unique first survey page visitors)	The recruitment rate was 90% (218 participants agreed to participate out of 242 invited participants who visited the first page)
	Completion rate (ratio of users who finished the survey/users who agreed to participate)	The completion rate was 91% (198 participants completed the survey out of 218 participants who agreed to participate)
**Preventing multiple entries from the same individual**		
	Cookies used	Cookies were not used to assign a unique user identifier; instead, we used users’ computer IP to identify unique users
	IP address check	The IP addresses of participants’ computers were used to identify potential duplicate entries from the same user. Only 2 duplicate entries were captured and were eliminated before analysis
	Log file analysis	We also checked the provided demographic information, of all participants, to make sure the 2 identified duplicates were the only incidents
	Registration	Data were collected and the user IP and other demographic data were used later on to eliminate duplicate entries before analysis. Most recent entries were used in the analysis
**Analysis**		
	Handling of incomplete questionnaires	Only completed surveys were used in the analysis
	Questionnaires submitted with an atypical timestamp	The task completion time was captured. However, no specific timeframe was used. In the analysis, we excluded statistical outliers, since the survey allowed users to re-enter after a while, for example, the next day. This is discussed in the paper
	Statistical correction	No statistical correction was required

^a^CDS: clinical decision support.

^b^IRB: institutional review board.

^c^IP: internet protocol.

## Results

### Descriptive Analysis

Out of 5857 relevant publications retrieved, 3282 professionals were identified and invited to participate in the survey. Over the survey duration of 6 weeks, from March 11 to April 21, 2019, we received a total of 194 valid responses, with a response rate of 5.9%. Valid responses were identified as those who completed the survey until the end and answered questions in all the survey sections, with no missing sections. Six participants missed answering one or more questions in one or more of the survey sections, 5 participants did not provide their demographics, and 57 participants did not wish to be acknowledged in the study. The detailed distributions of participants based on gender, age group, years of experience, clinical and health care role, clinical specialty, familiarity with head injury predictive tools, and their countries are illustrated in Multimedia Appendix 1, Figures 4-10.

### The GRASP Impact on Participants’ Decisions

Using the GRASP framework, an evidence-based summary of predictive tools and a detailed report on each predictive tool, along with allowing participants to search the internet for further information, made them select the correct tools 88.1% of the time. Without GRASP, that is, using the basic information about the predictive tools, the full text of the studies describing each tool, along with allowing participants to search the internet for further information, they selected the correct tools 53.7% of the time. This shows a statistically significant improvement of 64% (88.1/53.7=1.64; *P*<.001). On a 5-point Likert scale, where strongly agree is considered equal to 5 and strongly disagree is considered equal to 1, the participants reported that they made their tools’ selection decisions based on guessing with an average of 1.98 (SD 1.22), when they used GRASP, compared with an average of 2.48 (SD 1.37), when they did not use GRASP. This shows a statistically significant reduction of 20% (*P*<.001). Participants reported that they made their tools’ selection decisions based on their prior knowledge or experience with an average of 3.27 (SD 1.44) when they used GRASP, compared with an average of 3.55 (SD 1.31), when they did not use GRASP. This shows a statistically significant reduction of 8% (*P*=.004).

Participants reported that they made their tools’ selection decisions based on the information provided in the survey with an average of 4.10 (SD 1.10) when they used GRASP, compared with an average of 3.11 (SD 1.42), when they did not use GRASP. This shows a statistically significant increase of 32% (*P*<.001). Participants reported that they were confident in their decisions, with an average of 3.96 (SD 0.87), when they used GRASP, compared with an average of 3.55 (SD 1.15), when they did not use GRASP. This shows a statistically significant increase of 11% (*P*<.001). Participants reported that they were satisfied with their decisions with an average of 3.99 (SD 0.89), when they used GRASP, compared with an average of 3.54 (SD 1.20), when they did not use GRASP. This shows a statistically significant increase of 13% (*P*<.001). The duration of completing the task of selecting predictive tools showed high variability, with many statistical outliers. In addition to the average, the authors used the percentiles to avoid the effect of extreme outliers. The average duration of making the selection decisions showed a statistically insignificant reduction of 52% from 14.5 7 min (*P*=.39). There is also a reduction of 18.9% from 2.2 to 1.8 min on the 50th percentile, 37.3% from 5.3 to 3.3 min on the 75th percentile, 48% from 12.4 to 6.4 min on the 90th percentile, and 30.6% from 19.2 to 13.3 min on the 95th percentile. Table 3 shows the impact of using GRASP on the 7 measures: decision accuracy, guessing, subjective decisions, objective decisions, confidence in decisions, satisfaction with decisions, and task completion duration 90th percentile in minutes.

Using a paired samples *t* test, Table 4 shows the estimation for the paired difference of the 7 measures and the effect size, calculating and interpreting the eta-squared statistic, based on the guidelines proposed by Cohen [58].

Table 5 compares physicians to nonphysicians, emergency medicine to other specialties, familiar with tools to nonfamiliar, male to female, younger to older, and less experienced to more experienced participants. The GRASP detailed report is shown in Multimedia Appendix 1. The GRASP evidence-based summaries of the 2 groups of pediatric and adult predictive tools are shown in Multimedia Appendix 1.

**Table 3:** The impact of using grading and assessment of predictive tools on participants’ decisions (n=194).

Criteria	No GRASP^a^	GRASP	Change (%)	*P* value
Score (0 to 100%)	53.7	88.1	64	<.001
Guessing (1 to 5)	2.48	1.98	−20	<.001
Subjective (1 to 5)	3.55	3.27	−8	.003
Objective (1 to 5)	3.11	4.10	32	<.001
Confidence (1 to 5)	3.55	3.96	11	<.001
Satisfaction (1 to 5)	3.54	3.99	13	<.001
Time in min (90th percentile)	12.4	6.4	−48	.38

^a^GRASP: grading and assessment of predictive tools.

**Table 4:** Estimation for paired difference and effect size.

Measure	Mean (SD)	SE	99.3% CI^a^	*t* test (*df*)	*P* value	Effect size^b^			
						Value	Actual size		
Score	0.340 (0.555)	0.040	0.231 to 0.449	8.53 (193)	<.001	0.274	Large		
Guessing	−0.519 (1.303)	0.095	−0.777 to −0.260	−5.47 (188)	<.001	0.134	Moderate		
Subjective	−0.319 (1.464)	0.107	−0.613 to −0.028	−2.99 (187)	.003	0.044	Small		
Objective	1.005 (1.496)	0.109	0.709 to 1.302	9.24 (189)	<.001	0.307	Large		
Confidence	0.392 (1.261)	0.092	0.141 to 0.642	4.27 (188)	<.001	0.086	Moderate		
Satisfaction	0.439 (1.235)	0.090	0.194 to 0.684	4.89 (188)	<.001	0.110	Moderate		
Duration^c^	−447 (7152)	514	−1847 to 952	−0.87 (193)	.39	N/A^d^		N/A	

^a^Bonferroni correction conducted.

^b^Effect size calculated using the eta-square statistic (0.01=small effect, 0.06=moderate effect, and 0.14=large effect [58]).

^c^Task completion duration is reported in seconds.

^d^N/A: not applicable.

**Table 5:** Comparing the impact of grading and assessment of predictive tools on participant groups.

Health care professional group				Criteria												
				Score (0 to 100%)		Guessing (1 to 5)		Subjective (1 to 5)		Objective (1 to 5)		Confidence (1 to 5)		Satisfaction (1 to 5)		Time in min (90th percentile)
**Role**																
	**Physicians (n=130)**															
		No GRASP^a^	61.4		2.4		3.7		3.0		3.6		3.6		10.9	
		GRASP	89.0		2.0		3.5		4.0		4.0		4.0		6.1	
		Change (%)	45		−18		−5		33		10		12		−44	
		*P* value	<.001		<.001		.080		<.001		<.001		<.001		.62	
	**Nonphysicians (n=59)**															
		No GRASP	37		2.7		3.3		3.5		3.5		3.5		15.3	
		GRASP	85		2.0		2.8		4.4		3.8		3.9		6.6	
		Change (%)	127		−25		−16		28		10		14		−57	
		*P* value	<.001		<.001		.007		<.001		.047		.008		.26	
**Specialty**																
	**Emergency (n=94)**															
		No GRASP	73		2.4		4.1		2.8		3.8		3.8		11.0	
		GRASP	93		1.9		3.7		3.8		4.1		4.1		6.5	
		Change (%)	29		−19		−10		36		6		7		−41	
		*P* value	<.001		<.001		.009		<.001		.07		.04		.51	
	**Nonemergency (n=95)**															
		No GRASP	36		2.6		3.0		3.4		3.3		3.2		15.0	
		GRASP	83		2.0		2.9		4.4		3.8		3.8		6.5	
		Change (%)	129		−21		−6		28		15		19		−57	
		*P* value	<.001		<.001		.096		<.001		.001		<.001		.11	
**Familiarity with tools**																
	**Familiar (n=108)**															
		No GRASP	67.0		2.3		4.1		2.8		3.8		3.8		8.1	
		GRASP	89.6		1.8		3.7		3.8		4.1		4.1		5.3	
		Change (%)	34.0		−22.0		−10.0		39.0		8.0		8.0		−34.0	
		*P* value	<.001		<.001		.007		<.001		.016		.013		.51	
	**Not familiar (n=81)**															
		No GRASP	36		2.7		2.8		3.6		3.3		3.2		18.2	
		GRASP	85		2.2		2.7		4.5		3.7		3.8		7.9	
		Change (%)	134		−18		−5		23		14		19		−57	
		*P* value	<.001		.002		0.16		<.001		.003		<.001		.24	
**Gender**																
	**Males (n=120)**															
		No GRASP	54.2		2.3		3.5		3.1		3.7		3.6		13.5	
		GRASP	82.2		2.0		3.3		4.1		3.9		4.0		7.4	
		Change (%)	52		−14		−7		33		8		10		−45	
		*P* value	<.001		.005		.08		<.001		.009		.002		.41	
	**Females (n=67)**															
		No GRASP	55		2.9		3.5		3.3		3.3		3.4		12.2	
		GRASP	97		2.0		3.1		4.3		3.9		4.0		5.3	
		Change (%)	78		−30		−12		29		17		18		−56	
		*P* value	<.001		<.001		.004		<.001		.004		.001		.54	
**Age (years)**																
	**Younger (<45 years, n=112)**															
		No GRASP	59		2.6		3.6		3.1		3.5		3.5		9.1	
		GRASP	87		2.0		3.3		4.1		4.0		4.0		6.0	
		Change (%)	48		−25		−7		34		13		14		−34	
		*P* value	<.001		<.001		.06		<.001		.001		.001		.45	
	**Older (>45 years, n=77)**															
		No GRASP	47		2.3		3.5		3.2		3.6		3.6		15.9	
		GRASP	88		2.0		3.2		4.1		3.9		4.0		7.7	
		Change (%)	89		−13		−10		28		7		10		−52	
		*P* value	<.001		.03		.009		<.001		.08		.004		.19	
**Experience**																
	**Less experience (<15 years, n=94)**															
		No GRASP	59		2.6		3.6		3.0		3.5		3.4		8.1	
		GRASP	87		2.0		3.3		4.0		3.9		4.0		6.5	
		Change (%)	48		−24		−7		36		12		16		−20	
		*P* value	<.001		<.001		.09		<.001		.009		.001		.46	
	**More experience (>15 years, n=95)**															
		No GRASP	49		2.4		3.5		3.3		3.6		3.6		15.0	
		GRASP	88		2.0		3.2		4.2		3.9		4.0		6.8	
		Change (%)	80		−16		−10		28		9		9		−54	
		*P* value	<.001		.004		.006		<.001		.004		.004		.11	

^a^GRASP: grading and assessment of predictive tools.

### The GRASP Usability and Usefulness

The overall SUS rate of the GRASP framework and evidence-based summary, considering the responses of all 194 participants, was 72.5%, which represents a very good level of usability [59,60]. Examining the influence of demographics on the SUS rates, only 2 factors showed significant influence: the gender of participants and their familiarity with predictive tools. The female participants reported a statistically significant higher SUS rate (76.2%) in comparison with the male participants (70.8%), showing that female participants, more than male participants, thought GRASP is easy to use. Using the statistical Spearman correlation test, the degree of familiarity with head injury predictive tools showed a weak negative statistically significant correlation with the GRASP SUS score (*P*=.03). This indicates that participants who were less familiar with predictive tools thought that the GRASP framework was easy to use more than participants who were more familiar with the tools.

Among the 194 valid responses of participants, almost two-third (122) provided free-text feedback on the GRASP evidence-based summary usefulness and explained their feedback. The qualitative analysis of the open-ended question was conducted using the NVivo Version 12.3 software package [61]. Most respondents (88%, 108/122) reported that they found the GRASP evidence-based summary useful. They explained their responses with various reasons, mainly that the evidence-based summary was simple, clear, and logical. Some reported that the visual presentation was attractive, intuitive, and self-explanatory. Others reported that it concisely and comprehensively provided a summary of extensive information, and some reported that the presented information was consistent, easily comparable, making it easy to make informed decisions. A smaller group of 12% of participants reported that they found the GRASP evidence-based summary useless. They reported that it did not provide enough information to make informed decisions. Some reported that it was not clear enough, or simple enough, to understand and use to select predictive tools. One health care professional reported that “it is too complicated and needs to be simplified further,” while another reported that “it is oversimplified and missing some important parameters.” One health care professional reported “it might be more helpful when the decision is less clear” and added, “I would like to see more info on the strengths/weaknesses of each tool.”

## Discussion

### Brief Summary

The use of GRASP has positively supported, and significantly improved, evidence-based decision making and increased the accuracy and efficiency of selecting predictive tools. Using the GRASP framework has significantly increased correct decisions and objective decision making, and significantly decreased subjective decision making based on guessing, prior knowledge, or experience. Moreover, using the GRASP framework significantly decreased decisional conflict, increasing the confidence and satisfaction of participants with their decisions. Furthermore, using the GRASP framework decreased the task completion time for selecting predictive tools. In addition, the average SUS of the GRASP framework was very good, and most participants found the GRASP framework useful.

It is a challenging task for most health care professionals to critically evaluate a growing number of predictive tools, proposed in the literature, to select effective tools for implementation in clinical practice or for recommendation in clinical guidelines, to be used by other professionals. Although most of these predictive tools have been assessed for predictive performance, only a few have been implemented and evaluated for comparative effectiveness or postimplementation impact. Professionals need an evidence-based approach to provide them with standardized objective information on predictive tools to support their search for and selection of effective tools for clinical tasks. On the basis of the critical appraisal of the published evidence, the GRASP framework uses 3 dimensions to grade predictive tools: (1) phase of evaluation, (2) level of evidence, and (3) direction of evidence. The final grade assigned to a tool is based on the highest phase of evaluation, supported by the highest level of positive evidence, or mixed evidence that supports a positive conclusion. In this study, we present an evaluation of the impact of the GRASP framework on professionals’ decisions in selecting predictive tools for CDS.

### The Impact of GRASP on Participants’ Decisions

The GRASP framework provides a systematic and transparent approach for professionals to make objective, well-informed, and evidence-based decisions regarding the selection of predictive tools. This is very similar to the findings of using the Grading of Recommendations Assessment, Development and Evaluation (GRADE) framework in evaluating the quality of evidence and strength of recommendations regarding treatment methods and decisions endorsed in clinical guidelines [62,63]. The quality of decision making, while developing clinical guidelines, depends significantly on the quality of the evidence-informed analysis and advice provided [64]. Similarly, supporting professionals with evidence improves their accuracy and helps them make better clinical decisions and better organizational decisions [65,66]. Similarly, using GRASP and providing professionals with evidence-based information on predictive tools significantly improved professionals’ accuracy of decisions in selecting the best predictive tools.

Providing professionals with GRASP evidence-based information also enabled them to minimize subjective decision making, such as guessing, prior knowledge, or previous experience. This has been discussed in other studies investigating the role of utilizing evidence-based resources in decreasing subjective bias in making clinical, population-related, and health policy decisions [67,68]. Evidence-based information on GRASP was associated with a decrease in professionals’ decisional conflict by increasing their confidence in their decisions and their satisfaction with them. This has been discussed in similar studies reporting the impact of evidence-based information on decreasing decisional conflicts faced by both professionals and patients when they make clinical decisions [69-71]. When time is a sensitive factor for critical clinical and population decisions, efficient decision making becomes important [72]. Here comes the role of evidence-based decision making, which is discussed to be not only more accurate, objective, and of higher quality but also much more efficient [73,74]. Similarly, providing professionals with GRASP evidence-based information improved their efficiency in making predictive tools’ selection decisions.

Using GRASP made nurses and other professionals make more accurate decisions than physicians when they are not using GRASP. Using GRASP, clinicians of specialties other than emergency medicine make better decisions than emergency medicine clinicians without GRASP. Furthermore, using GRASP, professionals who were not familiar with head injury predictive tools made better decisions than professionals who were familiar with the tools without GRASP. Furthermore, the use of GRASP made decisions more efficient. Accordingly, using GRASP has moved professionals with less knowledge, less experience, and less familiarity with predictive tools to higher accuracy, higher efficiency, and better decision-making levels than professionals who had more knowledge, had more experience, and were more familiar with tools, but did not use GRASP.

### The Usability and Usefulness of GRASP

The usability of systems is an important foundation for successful implementation and utilization [75]. Usability can be evaluated by measuring the effectiveness of task management with accuracy and completeness, measuring the efficiency of utilizing resources in completing tasks and measuring users’ satisfaction, comfort with, and positive attitudes toward, the use of the tools [76,77]. One of the validated and simply applicable methods of measuring usability is the SUS [52,53]. When users have more experience with a system, they tend to provide higher, more favorable SUS scores for the system usability over users with either no or limited experience [78]. On the other hand, when users have less experience with a system, they tend to see new tools illustrating the system, or new approaches to understanding it, more usable than users who have extensive experience with the system itself [79]. This explains why the degree of familiarity with the tools was negatively correlated with the GRASP SUS score, where participants less familiar with tools provided higher SUS scores for GRASP than participants who were more familiar. It is reported in the literature that gender does not influence the perceived usability or usefulness of systems [80,81]. This was not the case with GRASP, where female participants provided higher SUS scores than males. Furthermore, female participants also thought that GRASP is more useful than males. Both findings could be explained by the greater improvement in female participants’ confidence and satisfaction with their decisions when they used GRASP compared with male participants. Some participants’ suggestions, reported in the free-text feedback, can be used in the future to add more information to the GRASP detailed report on each tool.

### Study Conclusions

Through this study, the GRASP framework is presented as an effective evidence-based approach to support professionals’ decisions when selecting predictive tools for implementation in clinical practice or for recommendation in clinical practice guidelines. Using the GRASP framework and the evidence-based summary improved the accuracy of selecting the best predictive tools, with an increased objective, informed, and evidence-based decision making and decreased subjective decision making based on guessing, prior knowledge, or experience. Using GRASP also decreased the decisional conflict faced by professionals by improving their confidence and satisfaction with their decisions. Using GRASP has also improved the efficiency of professionals in making their selection decisions by decreasing the time needed to complete the decision-making task.

The GRASP framework represents a high-level approach to provide professionals with an evidence-based and comprehensive, yet simple and feasible method to evaluate and select predictive tools. However, when professionals need further information, the detailed framework report provides them with the required details to support their decision making. The GRASP framework is designed for 2 levels of users:

(1) Expert users, such as health care researchers, experienced in evidence-based evaluation methods. They will use the framework to critically evaluate published evidence, assign grades to predictive tools, and report their details.

(2) End users, such as clinicians and health care professionals, responsible for selecting tools for implementation in clinical practice or for recommendation in clinical guidelines. They will use the GRASP framework detailed reports on tools and their assigned grades, produced by expert users, to compare existing predictive tools and select the most suitable tools [27].

The GRASP framework is not meant to be absolutely prescriptive. A lower grade tool could be preferred by a health care professional to improve clinical outcomes that are not supported by a higher grade one. For example, a practicing clinician may prefer an A2 tool showing improved patient safety in 2 observational studies rather than an A1 tool showing reduced health care costs in three experimental studies because they are now trying to improve patient safety to avoid reducing health care costs. It all depends on the objectives and priorities that the clinicians and health care professionals are trying to achieve. In addition, sometimes, more than one predictive tool should be endorsed in clinical practice guidelines, each supported by its requirements for application, conditions of use, and recommended for its most prominent outcomes of predictive performance or postimplementation impact on health care and clinical outcomes. Furthermore, even when GRASP assigns high grades to predictive tools, some of these tools may not be simply recommended for use in a different country or population than the ones that were used to develop and validate the tools in the first place. This might happen because of the population-related differences in the risks associated with the incidence of certain medical conditions, outcomes, or prognoses. This necessitates adjustment of the tools to the local context, thereby producing new versions of the tools, which requires re-evaluation by GRASP.

Although the GRASP framework has been developed to assess and grade predictive tools and other similar CDS systems, the application of the framework concept of grading tools and systems based on the published evidence is not limited to predictive tools or CDS systems. The GRASP framework concept can be applied to assess and grade many other types of clinical tools, systems, and methods.

### Study Limitations and Future Work

Although we received a large and sufficient number of 194 valid responses, the very low response rate of 5.9% could have been improved if potential participants were motivated by some incentives. They could have also been motivated if more support was provided through their organizations, which need more resources to synchronize such efforts. For the sake of keeping the survey feasible, for most busy professionals, the number of questions was kept limited and the time required to complete the survey was kept in the range of 20 min. However, some of the participants showed their willingness to provide more detailed feedback, which could have been done through interviews, for example, but this was out of the scope of the study and was not initially possible with the huge number of invited participants. The reduction in the decision-making duration of selecting predictive tools, while using GRASP, was statistically insignificant, because of the high variability and extreme statistical outliers, with and without GRASP. This could be explained by the fact that the Qualtrics platform of the survey measures the task completion duration by subtracting the time of loading the page from the time of pushing the Next button after completing the task and not the actual time the participants spent active on the page, which is currently under development [82].

To enable a wider global audience of clinicians, health care professionals, and clinical guideline developers to access detailed information, reported evidence, and assigned grades of different predictive tools, it is essential to implement the GRASP framework into a web-based platform. However, maintaining such a grading system up to date is a challenging task, as this requires continuous updating of the predictive tools grading and assessments when newly published evidence becomes available. In addition, the entire process is currently conducted manually, which represents a large burden on assessing and grading the huge number of existing predictive tools and those continuously emerging. Accordingly, it is essential to use automated or semiautomated methods for searching and processing new information to keep the GRASP framework information, grades, and assessments updated. Finally, we recommend that the GRASP framework be utilized by working groups of professional organizations to grade predictive tools to provide consistent results and increase reliability and credibility for end users. These professional organizations should also support disseminating such evidence-based information on predictive tools, similar to announcing and disseminating new updates of clinical practice guidelines.

## Appendix

Multimedia Appendix 1 The GRASP Framework detailed report.

Multimedia Appendix 2 CONSORT-eHEALTH checklist (V 1.6.1).

## Abbreviations

CCHR: Canadian CT Head Rule
CDS: clinical decision support
ED: emergency department
GRASP: grading and assessment of predictive tools
NOC: New Orleans Criteria
PECARN: Pediatric Emergency Care Applied Research Network
SUS: System Usability Scale
TBI: traumatic brain injury
