# Supplementary material for: Evaluating the Impact of the Grading and Assessment of Predictive Tools Framework on Clinicians and Health Care Professionals’ Decisions in Selecting Clinical Predictive Tools: Randomized Controlled Trial
Source: J Med Internet Res. 2020 Jul 9;22(7):e15770. doi: 10.2196/15770 (PMC7381257; doi:10.2196/15770)
Supplement: Multimedia Appendix 1 [file jmir_v22i7e15770_app1.pdf]

The Appendix

The GRASP Framework Detailed Report

Table 6: The GRASP Framework Detailed Report

<table>
<tr><td>Name</td><td colspan="3">Name of predictive tool (report tool’s creators and year in the absence of a given name)</td></tr>
<tr><td>Author</td><td colspan="3">Name of developer (first author or researcher)</td></tr>
<tr><td>Country</td><td colspan="3">Country of development</td></tr>
<tr><td>Year</td><td colspan="3">Year of development</td></tr>
<tr><td>Category</td><td colspan="3">Diagnostic/Therapeutic/Prognostic/Preventive</td></tr>
<tr><td>Intended use</td><td colspan="3">Specific aim/intended use of the predictive tool</td></tr>
<tr><td>Intended user</td><td colspan="3">Type of practitioner intended to use the tool</td></tr>
<tr><td>Clinical area</td><td colspan="3">Clinical specialty</td></tr>
<tr><td>Target Population</td><td colspan="3">Target patient population and health care settings in which the tool is applied</td></tr>
<tr><td>Target Outcome</td><td colspan="3">Event to be predicted (including prediction lead time if needed)</td></tr>
<tr><td>Action</td><td colspan="3">Recommended action based on tool’s output</td></tr>
<tr><td>Input source</td><td colspan="3">• Clinical (including Diagnostic, Genetic, Vital signs, Pathology)
• Non-Clinical (including Healthcare Utilisation)</td></tr>
<tr><td>Input type</td><td colspan="3">• Objective (Measured input; from electronic systems or clinical examination)
• Subjective (Patient reported; history, checklist …etc.)</td></tr>
<tr><td>Local context</td><td colspan="3">Is the tool developed using location-specific data? (e.g. life expectancy tables)</td></tr>
<tr><td>Methodology</td><td colspan="3">Type of algorithm used for developing the tool (e.g. parametric/non-parametric)</td></tr>
<tr><td>Internal Validation</td><td colspan="3">Method of internal validation</td></tr>
<tr><td>Dedicated Support</td><td colspan="3">Name of the supporting/funding research networks, programs, or professional groups</td></tr>
<tr><td>Endorsement</td><td colspan="3">Organisations endorsing the tool and/or clinical guidelines recommending its utilisation</td></tr>
<tr><td>Automation Flag</td><td colspan="3">Automation status (manual/automated)</td></tr>
<tr><td>Tool Citations</td><td colspan="3">Total citations of the tool</td></tr>
<tr><td>Studies</td><td colspan="3">Number of studies reporting the tool</td></tr>
<tr><td>Authors No</td><td colspan="3">Number of authors</td></tr>
<tr><td>Sample Size</td><td colspan="3">Size of patient/record sample used in the development of the tool</td></tr>
<tr><td>Journal Name</td><td colspan="3">Name of the journal that published the tool’s primary development study</td></tr>
<tr><td>Journal Rank</td><td colspan="3">Impact factor of the journal</td></tr>
<tr><td>Citation Index</td><td colspan="3">Calculated as: Average Annual Citations = number of citations/age of primary publication</td></tr>
<tr><td>Publication Index</td><td colspan="3">Calculated as: Average Annual Studies = number of studies/age of primary publication</td></tr>
<tr><td>Literature Index</td><td colspan="3">Calculated as: Citations and Publications = number of citations X number of studies</td></tr>
<tr><td>Phase of Evaluation</td><td>Level of Evidence</td><td>Grade</td><td>Evaluation Studies</td></tr>
<tr><td rowspan="4">Phase C:

Before implementation

Is it possible?</td><td>Insufficient internal validation</td><td>C0</td><td>Not tested for internal validity, insufficiently internally validated, or internal validation was insufficiently reported.</td></tr>
<tr><td>Internal validation</td><td>C3</td><td>Tested for internally validity (reported calibration & discrimination; sensitivity, specificity, positive and negative predictive values & other predictive performance measures).</td></tr>
<tr><td>External validation</td><td>C2</td><td>Tested for external validity, using one external dataset.</td></tr>
<tr><td>External validation multiple times</td><td>C1</td><td>Tested multiple times for external validity, using more than one external dataset.</td></tr>
</table>

<table>
<tr><td rowspan="3">Phase B:

Planning for implementation

Is it practicable?</td><td>Usability</td><td>B3</td><td colspan="9">Reported usability testing (tool effectiveness, efficiency, satisfaction, learnability, memorability, and minimizing errors).</td></tr>
<tr><td>Potential effect</td><td>B2</td><td colspan="9">Reported estimated potential effect on clinical effectiveness, patient safety or healthcare efficiency.</td></tr>
<tr><td>Potential effect & Usability</td><td>B1</td><td colspan="9">Both potential effect and usability are reported.</td></tr>
<tr><td rowspan="3">Phase A:

After implementation:

Is it desirable?</td><td rowspan="3">Evaluation of post-implementation impact on Clinical Effectiveness, Patient Safety or Healthcare Efficiency</td><td>A3</td><td colspan="9">Based on subjective studies; e.g. the opinion of a respected authority, clinical experience, a descriptive study, or a report of an expert committee or panel.</td></tr>
<tr><td>A2</td><td colspan="9">Based on observational studies; e.g. a well-designed cohort or case-control study.</td></tr>
<tr><td>A1</td><td colspan="9">Based on experimental studies; properly designed, widely applied randomised/nonrandomised controlled trial.</td></tr>
<tr><td>Assigned Grade</td><td colspan="2">Grade ABC/123</td><td>A1</td><td>A2</td><td>A3</td><td>B1</td><td>B2</td><td>B3</td><td>C1</td><td>C2</td><td>C3</td></tr>
<tr><td rowspan="2">Direction of Evidence</td><td colspan="2">● Positive Evidence</td><td colspan="9">◕ Mixed Evidence Supporting Positive Conclusion</td></tr>
<tr><td colspan="2">○ Negative Evidence</td><td colspan="9">◔ Mixed Evidence Supporting Negative Conclusion</td></tr>
<tr><td>Justification</td><td colspan="11">Explains how the final grade is assigned based on evidence; which conclusions were taken into consideration, as positive evidence, and which were considered negative.</td></tr>
<tr><td>References</td><td colspan="11">Details of studies that support the justification: phase of evaluation, level of evidence, direction of evidence, study type, study settings, methodology, results, findings and conclusions (highlighted according to the findings codes).</td></tr>
<tr><td>Findings Codes</td><td colspan="11">Positive Findings / Negative Findings / Important Findings</td></tr>
</table>

The GRASP Evidence Based Summary of Predictive Tools

Table 7: The GRASP Evidence-Based Summary of Paediatric Head Injury Predictive Tools

Tool	Tool Information				Tool Grade	Impact After Implementation			Planning for Implementation			Performance Before Implementation		
	Country	Year	Citations	Studies		Experimental Studies	Observational Studies	Subjective Studies	Potential Effect & Usability	Potential Effect	Usability	External Validation Multiple Times	External Validation Only Once	Internal Validation
						A1	A2	A3	B1	B2	B3	C1	C2	C3
**PECARN**	USA	2009	886	24	**A2**		◕			●		●		●
**CHALICE**	UK	2006	308	15	**B2**					◕		●		●
**CATCH**	USA	2006	321	12	**C1**							●		●
**Palchak**	USA	2003	247	3	**C2**								●	●
**Atabaki**	USA	2008	111	1	**C3**									●
**Evidence Direction**	● Positive Evidence					◕ Mixed Evidence Supporting Positive Conclusion								
	○ Negative Evidence					◔ Mixed Evidence Supporting Negative Conclusion								

Table 8: The GRASP Evidence-Based Summary of Adult Head Injury Predictive Tools

Tool	Tool Information				Tool Grade	Impact After Implementation			Planning for Implementation			Performance Before Implementation		
	Country	Year	Citations	Studies		Experimental Studies	Observational Studies	Subjective Studies	Potential Effect & Usability	Potential Effect	Usability	External Validation Multiple Times	External Validation Only Once	Internal Validation
						A1	A2	A3	B1	B2	B3	C1	C2	C3
**CCHR**	Canada	2001	1098	23	**C1**	○	○			○		●		●
**NOC**	USA	2000	907	11	**C1**					○		●		●
**Miller**	USA	1997	210	2	**C3**								○	●
**KHR**	S. Africa	2013	7	1	**C3**									●
**Ibanez**	Spain	2004	165	1	**C0**									○
**Evidence Direction**	● Positive Evidence					◕ Mixed Evidence Supporting Positive Conclusion								
	○ Negative Evidence					◔ Mixed Evidence Supporting Negative Conclusion								

Statistical Tables and Figures

Table 9: Gender Distribution of Participants

Gender	Count	Percentage	Cum
Males	120	61.9%	61.9%
Females	67	34.5%	96.4%
Not Reported	7	3.6%	100.0%
Total	**194**	**100%**	

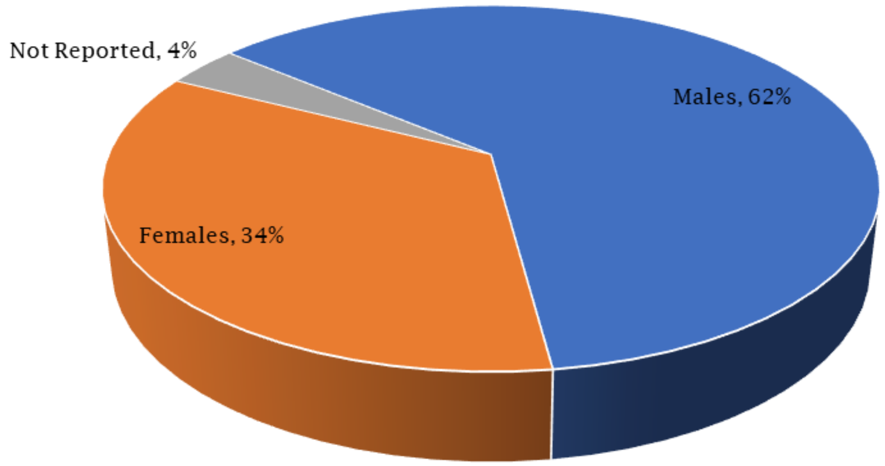

Figure 4: Gender Distribution of Participants

Table 10: Age Group Distribution of Participants

Age Group	Count	Percentage	Cum
25 – 34 years	23	11.9%	11.9%
35 - 44 years	89	45.9%	57.7%
45 - 54 years	49	25.3%	83.0%
55 - 64 years	20	10.3%	93.3%
65 - 74 years	7	3.6%	96.9%
75 years or older	1	0.5%	97.4%
Not Reported	5	2.6%	100.0%
**Total**	**194**	**100%**	
**Average Age**	**44.8 years**		

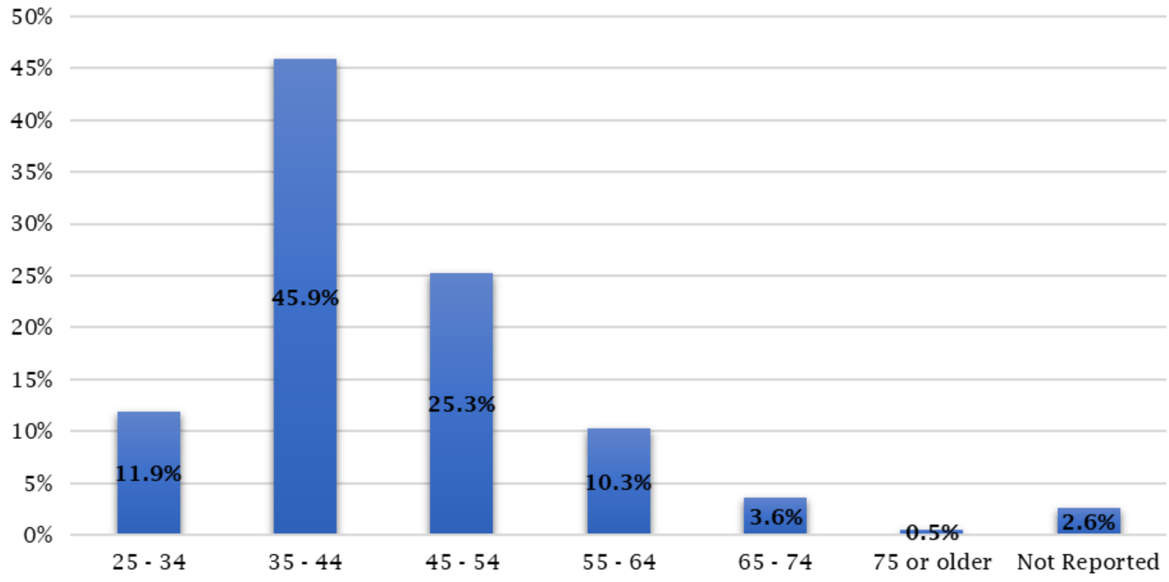

Figure 5: Age Group Distribution of Participants

Table 11: Years of Experience Distribution of Participants

Years of Experience	Count	Percentage	Cum
Less than 5 years	12	6.2%	6.2%
05-09 years	29	14.9%	21.1%
10-14 years	53	27.3%	48.5%
15-19 years	32	16.5%	64.9%
20-24 years	22	11.3%	76.3%
25-29 years	19	9.8%	86.1%
30-34 years	12	6.2%	92.3%
35 years or more	10	5.2%	97.4%
Not Reported	5	2.6%	100.0%
**Total**	**194**	**100%**	
**Average Age**	**16.7 years**		

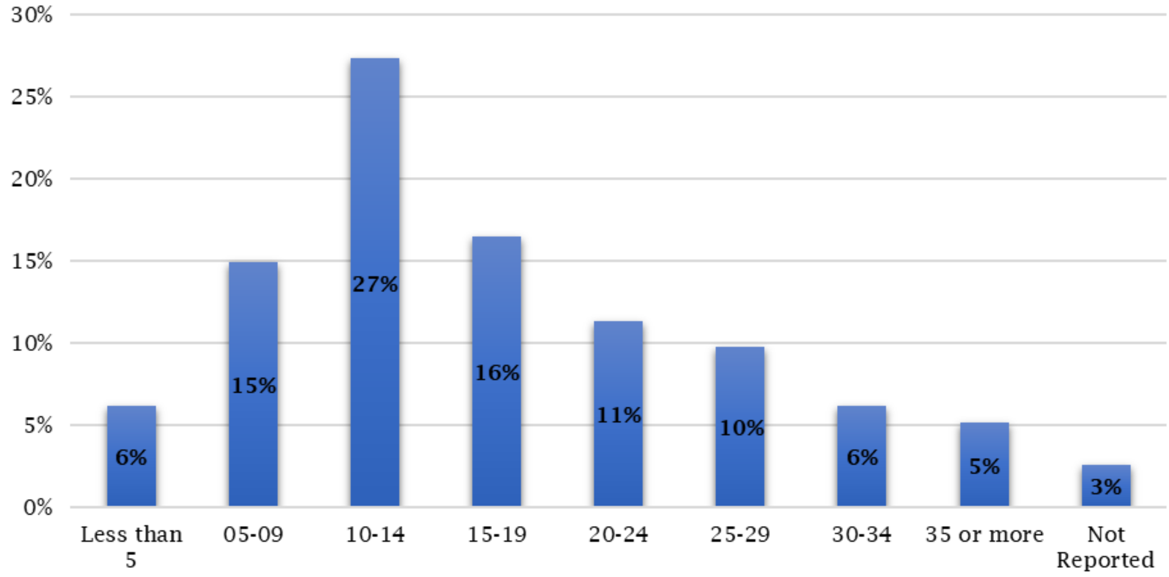

Figure 6: Years of Experience Distribution of Participants

Table 12: Role Distribution of Participants

Clinical Role	Count	Percentage	Cum
Physician - Consultant	114	58.8%	58.8%
Physician - Registrar	10	5.2%	63.9%
Physician - Resident	6	3.1%	67.0%
Nurse - Senior	13	6.7%	73.7%
Nurse - Junior	0	0.0%	73.7%
Other Healthcare professionals	46	23.7%	97.4%
Not Reported	5	2.6%	100.0%
**Total**	**194**	**100%**	

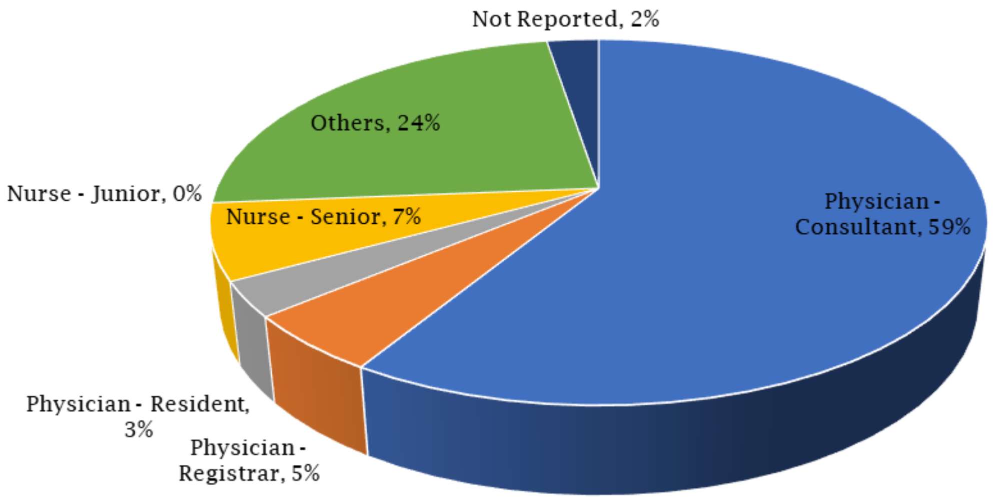

Figure 7: Role Distribution of Participants

Table 13: Specialty Distribution of Participants

Clinical Specialty	Count	Percentage	Cum
Emergency Medicine	94	48.5%	48.5%
Other Specialties	95	49.0%	97.4%
Not Reported	5	2.6%	100.0%
**Total**	**194**	**100%**	

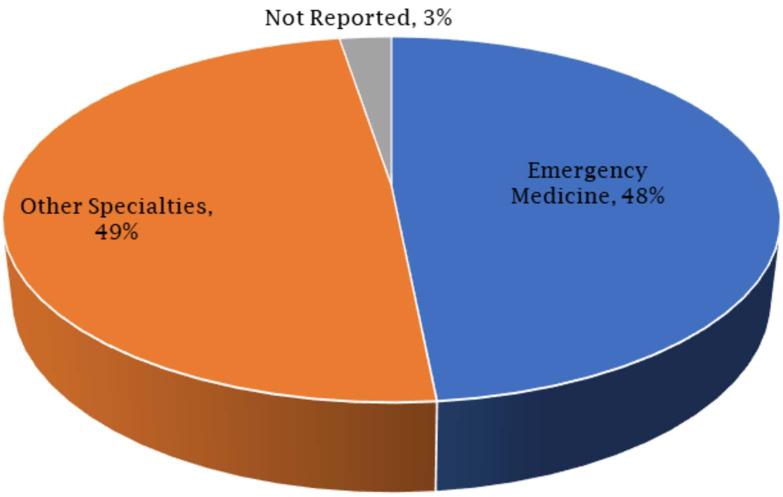

Figure 8: Specialty Distribution of Participants

Table 14: Distribution of Participants Who are Familiar with Predictive Tools

Familiar with Tools	Count	Percentage	Cum
Strongly Agree (code = 5)	47	24.2%	24.2%
Somewhat Agree (code = 4)	61	31.4%	55.7%
Neither Agree nor Disagree (code = 3)	18	9.3%	64.9%
Somewhat Disagree (code = 2)	33	17.0%	82.0%
Strongly Disagree (code = 1)	30	15.5%	97.4%
Not Reported	5	2.6%	100.0%
**Total**	**194**	**100%**	
**Average**	**Neither Agree nor Disagree (score = 3.33)**		

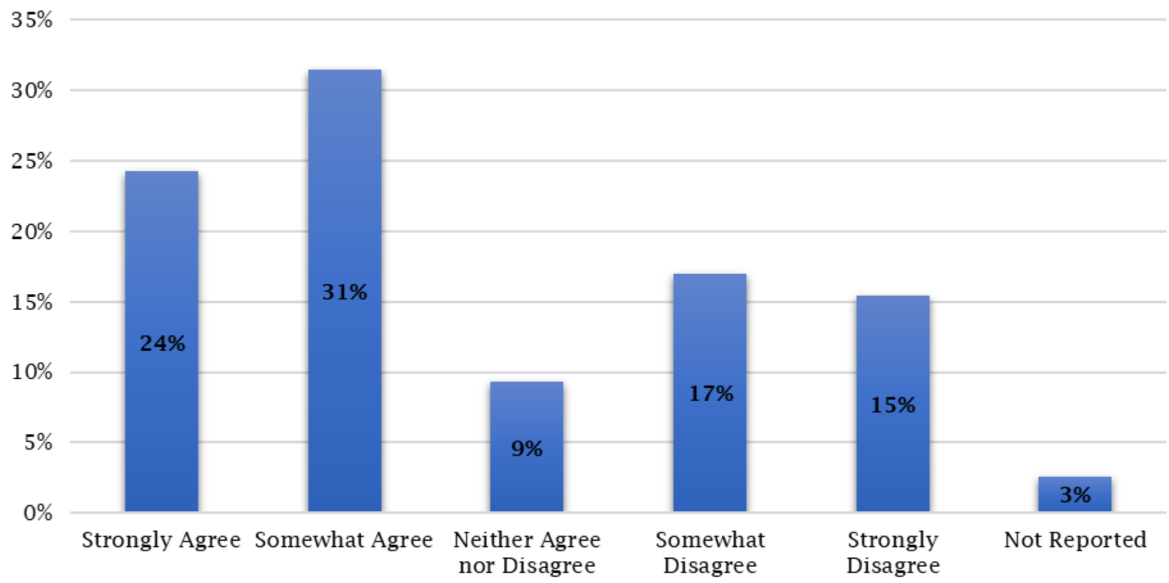

Figure 9: Distribution of Participants Who are Familiar with Predictive Tools

Table 15: Country Distribution of Participants

SN	Country	Respondents	Percent	Cum
1	United States	57	29.4%	29.4%
2	Australia	23	11.9%	41.2%
3	Italy	17	8.8%	50.0%
4	Canada	11	5.7%	55.7%
5	United Kingdom	11	5.7%	61.3%
6	Germany	6	3.1%	64.4%
7	Netherlands	5	2.6%	67.0%
8	Switzerland	5	2.6%	69.6%
9	Belgium	4	2.1%	71.6%
10	Saudi Arabia	4	2.1%	73.7%
11	Spain	4	2.1%	75.8%
12	Sweden	4	2.1%	77.8%
13	Brazil	3	1.5%	79.4%
14	China	3	1.5%	80.9%
15	Turkey	3	1.5%	82.5%
16	Colombia	2	1.0%	83.5%
17	India	2	1.0%	84.5%
18	Japan	2	1.0%	85.6%
19	Lebanon	2	1.0%	86.6%
20	Lithuania	2	1.0%	87.6%
21	Palestine	2	1.0%	88.7%
22	Poland	2	1.0%	89.7%
23	Portugal	2	1.0%	90.7%
24	Taiwan	2	1.0%	91.8%
25	Europe	6	3.1%	94.8%
26	Asia	4	2.1%	96.9%
27	Africa	3	1.5%	98.5%
28	South America	3	1.5%	100.0%
**Total**		**194**	**100%**	

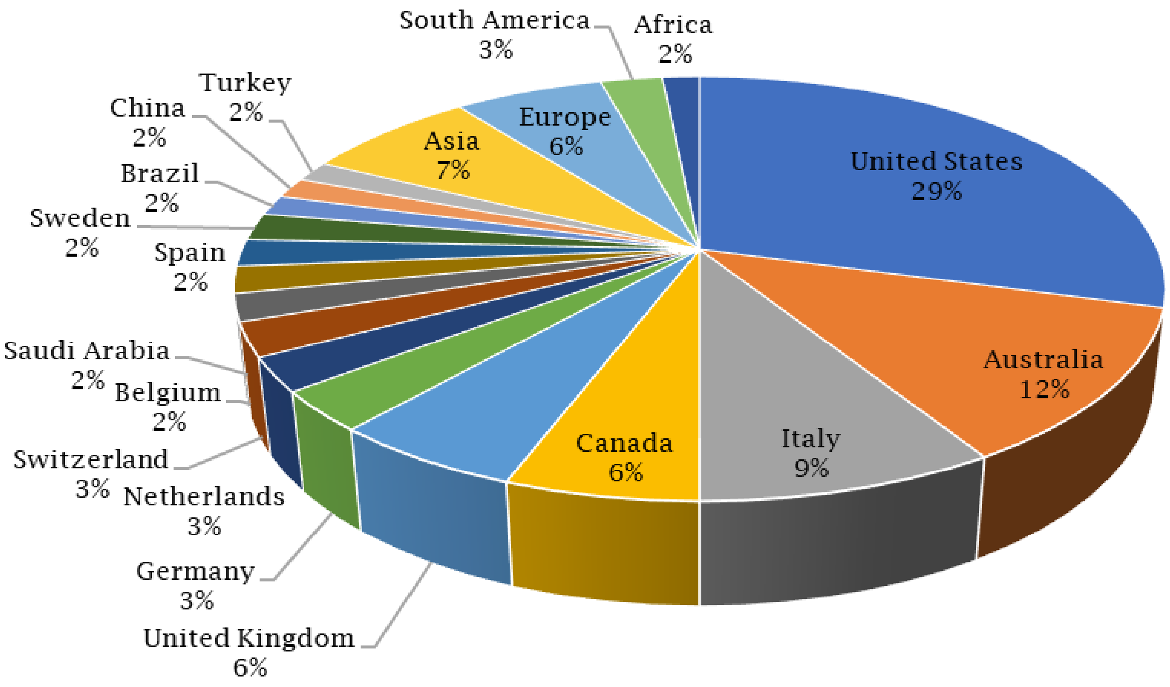

Figure 10: Country Distribution of Participants

The Survey Screenshots

MACQUARIE University

Validating a New Evidence-Based Framework for Evaluating Clinical Predictive Tools used in the Emergency Department

Background:

Clinical predictive tools are applications that provide diagnostic, prognostic, or therapeutic decision support. When selecting a predictive tool, for implementation at their clinical practice or for recommendation in clinical guidelines, clinicians involved in the decision making are challenged with an overwhelming and ever-growing number of tools. Most of these tools have never been implemented or assessed for comparative effectiveness.

The Framework:

This new framework grades clinical predictive tools based on the critical appraisal of the published evidence about the tools across three dimensions: 1) Phase of Evaluation (before, during and after implementation); 2) Level of Evidence (using a numerical score within each phase); and 3) Direction of Evidence (positive, negative, or mixed, based on the collective conclusions of the published studies about the tools). The final grade assigned to a predictive tool is based on the highest phase of evaluation, supported by the highest level of positive evidence, or mixed evidence that supports a positive conclusion.

The Task:

Based on the provided information in this survey, you are kindly requested to answer simple questions about clinical predictive tools used in the emergency department, with and without using the new framework. This will take around 20 minutes of your time. We highly appreciate your participation and feedback. This study has been approved by Macquarie University Human Research Ethics. All your answers will be kept confidential and will only be used for research.

Feedback and Acknowledgment:

After you complete this survey, you will have the option to be informed of the results and to be acknowledged in the publication of this research.

For further information, please contact:

Dr. Mohamed Khalifa
Australian Institute of Health Innovation
Macquarie University, 75 Talavera Rd, North Ryde, Sydney, NSW 2113, Australia
M: +61 438 632 060 | E: mohamed.khalifa@mq.edu.au

Research Chief Investigator:

A/Prof Blanca Gallego
Australian Institute of Health Innovation
Macquarie University, 75 Talavera Rd, North Ryde, Sydney, NSW 2113, Australia
T: +61 (02) 9850 1608 | E: blanca.gallegoluxan@mq.edu.au

For any complaints about ethical aspects of the research, you can contact:

Ms Vanessa Cooper
Ethics Officer, Australian Institute of Health Innovation
17 Wally's Walk, Level 3, Macquarie University, North Ryde, NSW 2113, Australia
T: +61 (02) 9850 2326 | E: vanessa.cooper@mq.edu.au

→

Section 1: The survey introduction

MACQUARIE University

Paediatric Head Injury Predictive Tools - Evidence-Based Summary

Assume you are the head of a busy emergency department and you are responsible for choosing a predictive tool, for the diagnosis of paediatric head injury, to be implemented at your department or to be recommended in the clinical guidelines. You have a list of five tools and you have an Evidence-Based Summary about the tools:

- **PECARN Rule (Paediatric Emergency Care Applied Research Network)**
- **CHALICE Rule (Children's Head injury ALgorithm for the prediction of Important Clinical Events)**
- **CATCH Rule (Canadian Assessment of Tomography for Childhood Head injury)**
- **Palchak (UC Davis) Rule for Paediatric Head Trauma**
- **Atabaki Rule for Paediatric Head Injury/Trauma**

Tool	Tool Information				Tool Grade	Impact After Implementation			Planning for Implementation			Performance Before Implementation		
	Country	Year	Citations	Studies		Experimental Studies A1	Observational Studies A2	Subjective Studies A3	Potential Effect & Usability B1	Potential Effect B2	Usability B3	External Validation Multiple Times C1	External Validation Only Once C2	Internal Validation C3
**PECARN**	USA	2009	886	24	**A2**		◕			●		●		●
**CHALICE**	UK	2006	308	15	**B2**					◕		●		●
**CATCH**	USA	2006	321	12	**C1**							●		●
**Palchak**	USA	2003	247	3	**C2**								●	●
**Atabaki**	USA	2008	111	1	**C3**									●
**Evidence Direction**	● Positive Evidence					◕ Mixed Evidence Supporting Positive Conclusion								
	○ Negative Evidence					◔ Mixed Evidence Supporting Negative Conclusion								

You can download the full evidence-based report on the five tools from this link: **Full Report**

Which predictive tool would you choose?

PECARN	CHALICE	CATCH	Palchak	Atabaki	I don't know
○	○	○	○	○	○

Regarding my decision in selecting this tool:

	Strongly agree	Somewhat agree	Neither agree nor disagree	Somewhat disagree	Strongly disagree
I made this decision based on guessing.	○	○	○	○	○
I made this decision based on my knowledge or experience	○	○	○	○	○
I made this decision based on the information provided in this survey.	○	○	○	○	○
I am confident in my decision.	○	○	○	○	○
I am satisfied with my decision.	○	○	○	○	○

→

Section 2: Block 1: Paediatric Head Injury Predictive Tools With GRASP

MACQUARIE University

Adult Head Injury Predictive Tools - Briefing Information

Assume you are the head of a busy emergency department and you are responsible for choosing a predictive tool, for the diagnosis of adult head injury, to be implemented at your department or to be recommended in the clinical guidelines. You have a list of five tools and you have briefing information about each tool. You can also download the original study of each tool form the links.

CCHR – The Canadian CT Head Rule

The tool was developed by Dr. Ian Stiell in Canada in 2001. The tool predicts the need for brain CT imaging in adults after minor head injury/trauma. This diagnostic tool is designed to be used at the emergency department by physicians to decide which patients need CT scan and/or acute intervention. Download the study: CCHR

Ibañez Model for Head CT

The tool was developed by Dr. Javier Ibañez in Spain in 2004. The tool predicts the need for brain CT imaging in adults after minor head injury/trauma. This diagnostic tool is designed to be used at the emergency department by physicians to decide which patients need CT scan and/or acute intervention. Download the study: Ibañez Model

KHR – The Kimberley Hospital Rule

The tool was developed by Dr. Abraham Bezuidenhout in South Africa in 2013. The tool predicts the need for brain CT imaging to diagnose trauma-related and non-trauma related acute intracranial pathology conditions in resource-limited environments. This diagnostic tool is designed to be used at the emergency department by physicians to decide which patients need CT scan and/or acute intervention. Download the study: KHR

Miller Criteria for Head CT

The tool was developed by Dr. Erik Miller in the United States in 1997. The tool predicts the need for brain CT imaging to diagnose minor head trauma in adults. This diagnostic tool is designed to be used at the emergency department by physicians to decide which patients need CT scan and/or acute intervention. Download the study: Miller Criteria

NOC – New Orleans Criteria

The tool was developed by Dr. Micelle Haydel in the United States in 2000. The tool predicts the need for brain CT imaging after adult head injury/trauma. This diagnostic tool is designed to be used at the emergency department by physicians to decide which patients need CT scan and/or acute intervention. Download the study: NOC

Which predictive tool would you choose?

CCHR	Ibanez	KHR	Miller	NOC	I don't know
○	○	○	○	○	○

Regarding my decision in selecting this tool:

	Strongly agree	Somewhat agree	Neither agree nor disagree	Somewhat disagree	Strongly disagree
I made this decision based on guessing.	○	○	○	○	○
I made this decision based on my knowledge or experience.	○	○	○	○	○
I made this decision based on the information provided in this survey.	○	○	○	○	○
I am confident in my decision.	○	○	○	○	○
I am satisfied with my decision.	○	○	○	○	○

← →

Section 2: Block 2: Adult Head Injury Predictive Tools Without GRASP

MACQUARIE University

Paediatric Head Injury Predictive Tools - Briefing Information

Assume you are the head of a busy emergency department and you are responsible for choosing a predictive tool, for the diagnosis of paediatric head injury, to be implemented at your department or to be recommended in the clinical guidelines. You have a list of five tools and you have briefing information about each tool. You can also download the original study of each tool form the links.

Atabaki Rule for Paediatric Head Injury

The tool was developed by Dr. Shireen Atabaki in the United States in 2008. The tool identifies children at low risk of brain injury after minor head trauma. This diagnostic tool is designed to be used at the emergency department by physicians to decide which patients need CT scan and/or acute intervention. Download the study: Atabaki Rule

CATCH Rule (Canadian Assessment of Tomography for Childhood Head injury)

The tool was developed by Dr. Martin Osmond in the United States in 2010. The tool predicts clinically significant head injury in children after minor head trauma. This diagnostic tool is designed to be used at the emergency department by physicians to decide which patients need CT scan and/or acute intervention. Download the study: CATCH Rule

CHALICE Rule (Children's Head injury ALgorithm for the prediction of Important Clinical Events)

The tool was developed by Dr. Joel Dunning in the United Kingdom in 2006. The tool predicts death, need for neurosurgical intervention or CT abnormality in children with minor head trauma. This diagnostic tool is designed to be used at the emergency department by physicians to decide which patients need CT scan and/or acute intervention. Download the study: CHALICE Rule

Palchak (UC Davis) Rule for Paediatric Head Trauma

The tool was developed by Dr. Michael Palchak and Dr. Nathan Kuppermann in the United States in 2003. The tool identifies children at low risk of brain injury after minor head trauma. This diagnostic tool is designed to be used at the emergency department by physicians to decide which patients need CT scan and/or acute intervention. Download the study: Palchak Rule

PECARN Rule (Paediatric Emergency Care Applied Research Network)

The tool was developed by Dr. Nathan Kuppermann in the United States in 2009. The tool identifies children at very low risk of clinically important brain injury after minor head trauma. This diagnostic tool is designed to be used at the emergency department by physicians to decide which patients need CT scan and/or acute intervention. Download the study: PECARN Rule

Which predictive tool would you choose?

Atabaki	CATCH	CHALICE	Palchak	PECARN	I don't know
○	○	○	○	○	○

Regarding my decision in selecting this tool:

	Strongly agree	Somewhat agree	Neither agree nor disagree	Somewhat disagree	Strongly disagree
I made this decision based on guessing.	○	○	○	○	○
I made this decision based on my knowledge or experience.	○	○	○	○	○
I made this decision based on the information provided in this survey.	○	○	○	○	○
I am confident in my decision.	○	○	○	○	○
I am satisfied with my decision.	○	○	○	○	○

→

Section 2: Block 3: Paediatric Head Injury Predictive Tools Without GRASP

MACQUARIE University

Adult Head Injury Predictive Tools - Evidence-Based Summary

Assume you are the head of a busy emergency department and you are responsible for choosing a predictive tool, for the diagnosis of adult head injury, to be implemented at your department or to be recommended in the clinical guidelines. You have a list of five tools and you have an Evidence-Based Summary about the tools:

- **CCHR – The Canadian CT Head Rule**
- **NOC – New Orleans Criteria**
- **Miller Criteria for Head CT**
- **KHR – The Kimberley Hospital Rule**
- **Ibanez Model for Head CT**

Tool	Tool Information				Tool Grade	Impact After Implementation			Planning for Implementation			Performance Before Implementation		
	Country	Year	Citations	Studies		Experimental Studies	Observational Studies	Subjective Studies	Potential Effect & Usability	Potential Effect	Usability	External Validation Multiple Times	External Validation Only Once	Internal Validation
						A1	A2	A3	B1	B2	B3	C1	C2	C3
CCHR	Canada	2001	1098	23	C1	○	○			○		●		●
NOC	USA	2000	907	11	C1					○		●		●
Miller	USA	1997	210	2	C3								○	●
KHR	S.Africa	2013	7	1	C3									●
Ibanez	Spain	2004	165	1	C0									○
Evidence Direction	● Positive Evidence					◔ Mixed Evidence Supporting Positive Conclusion								
	○ Negative Evidence					◔ Mixed Evidence Supporting Negative Conclusion								

You can download the full evidence-based report on the five tools from this link: **Full Report**

Which predictive tool would you choose?

CCHR	NOC	Miller	KHR	Ibanez	I don't know
○	○	○	○	○	○

Regarding my decision in selecting this tool:

	Strongly agree	Somewhat agree	Neither agree nor disagree	Somewhat disagree	Strongly disagree
I made this decision based on guessing.	○	○	○	○	○
I made this decision based on my knowledge or experience.	○	○	○	○	○
I made this decision based on the information provided in this survey.	○	○	○	○	○
I am confident in my decision.	○	○	○	○	○
I am satisfied with my decision.	○	○	○	○	○

← →

Section 2: Block 4: Adult Head Injury Predictive Tools With GRASP

MACQUARIE University

The Usability of the Evidence-Based Summary

Regarding the Evidence-Based Summary displayed in the previous screen: How much do you agree with each of the following:

	Strongly agree	Somewhat agree	Neither agree nor disagree	Somewhat disagree	Strongly disagree
I think I would like to use the Evidence-Based Summary frequently.	○	○	○	○	○
I found the Evidence-Based Summary unnecessarily complex.	○	○	○	○	○
I think the Evidence-Based Summary was easy to use.	○	○	○	○	○
I think I need the support of an expert person to be able to use the Evidence-Based Summary.	○	○	○	○	○
I found the functions in the Evidence-Based Summary well integrated.	○	○	○	○	○
I think there was too much inconsistency in the Evidence-Based Summary.	○	○	○	○	○
I think most people would learn to use the Evidence-Based Summary very quickly.	○	○	○	○	○
I found the Evidence-Based Summary very difficult to use.	○	○	○	○	○
I felt very confident using the Evidence-Based Summary.	○	○	○	○	○
I need to learn a lot of things before I can use the Evidence-Based Summary.	○	○	○	○	○

Do you find the evidence-based summary useful? And why?

← →

Section 3: GRASP System Usability Scale and Usefulness

MACQUARIE University

Demographic Information

My role in the hospital is best described as:

- ○ Physician – Consultant
- ○ Physician – Registrar
- ○ Physician – Resident
- ○ Nurse – Senior
- ○ Nurse – Junior
- ○ Other roles

My specialty is:

- ○ Emergency medicine / department
- ○ Other specialties / services

My gender is:

- ○ Male
- ○ Female
- ○ I prefer not to say

My age is:

- ○ Less than 25
- ○ 25 - 34
- ○ 35 - 44
- ○ 45 - 54
- ○ 55 - 64
- ○ 65 - 74
- ○ 75 or older

My experience in years:

- ○ Less than 5
- ○ 5 - 9
- ○ 10 - 14
- ○ 15 - 19
- ○ 20 - 24
- ○ 25 - 29
- ○ 30 - 34
- ○ 35 or more

I am familiar with the head injury clinical predictive tools:

- ○ Strongly agree
- ○ Somewhat agree
- ○ Neither agree nor disagree
- ○ Somewhat disagree
- ○ Strongly disagree

← →

Section 4: Participants' Demographics

MACQUARIE University

Feedback and Acknowledgement:

- [ ] I would like to receive a feedback on the results of this survey.
- [ ] I would like to be acknowledged in the publication of this study.

My name is:

My email is:

All your answers will be kept confidential and will only be used for the purposes of research. Your information is collected only to give you a feedback and will be kept confidential and separate from your answers.

For further information, please contact:

Dr. Mohamed Khalifa
Australian Institute of Health Innovation
Macquarie University, 75 Talavera Rd, North Ryde, Sydney, NSW 2113, Australia
M: +61 438 632 060 | E: mohamed.khalifa@mq.edu.au

Research Chief Investigator:

A/Prof Blanca Gallego
Australian Institute of Health Innovation
Macquarie University, 75 Talavera Rd, North Ryde, Sydney, NSW 2113, Australia
T: +61 (02) 9850 1608 | E: blanca.gallegoluxan@mq.edu.au

For any complaints about ethical aspects of the research, you can contact:

Ms Vanessa Cooper
Ethics Officer, Australian Institute of Health Innovation
17 Wally's Walk, Level 3, Macquarie University, North Ryde, NSW 2113, Australia
T: +61 (02) 9850 2326 | E: vanessa.cooper@mq.edu.au

← →

Section 5: Participants' Feedback and Acknowledgment
